# Minutes of the Wabash Valley Dental Association

**Published:** 1871-06

**Authors:** Seneca B. Brown


### Proceedings of Societies.

### MINUTES OF THE WABASH VALLEY DENTAL ASSOCIATION.

The Wabash Valley Dental Association convened for its seventh annual session in the rooms of the Y. M. C. A., at Lafayette, Ind., on Tuesday, March 21, 1871. The President, John L. Scott, in the chair. W. H. Pifer, Secretary.

The meeting was called to order at 10 o'clock, A. M., and upon the calling of the roll the following members answered to their names:

John L. Scott, Defiance, Ohio; A. B. Cunningham, Attica; A. M. Moore, Lafayette; Isaac Knapp, Fort Wayne; J. S. Snoddy, Lafayette; W. F. Lewis, Delphi; W. H. Pifer, Lafayette; Lewis Jourdan, Delphi; E. V. Burt, Lafayette; J. W. Fahnestock, Lafayette; Seneca B. Brown, Fort Wayne.

The minutes of the last meeting were read and approved.

The treasurer, J. S. Snoddy, having made a report, on motion action was deferred till afternoon session.

The Board of Censors being absent, the chair appointed A. B. Cunningham, A. M. Moore and Isaac Knapp in their stead.

The Board recommended Dr. E. Snider, of Fort Wayne, for membership. The rules being suspended, he was declared a member of the association.

The Treasurer being absent, Prof. A. M. Moore was appointed Treasurer *pro tem.*

Dr. E. V. Burt moved that Prof. Chase, of St. Louis, Dr. C. Palmer, of Warren, Ohio, Dr. W. Taft, of Cincinnati, and Dr. P. G. C. Hunt, of Indianapolis, be elected honorary members. Carried.

On motion the association proceeded to elect officers for the ensuing year, which resulted as follows:

President—A. B. Cunningham, Attica.

1st Vice President—E. Snider, Ft. Wayne.

2d Vice President—W. F. Lewis, Delphi.

Secretary—Seneca B. Brown, Fort Wayne.

Treasurer—A. M. Moore, Lafayette.

Dr. Burt moved to adjourn till 2 P. M.

Dr. Moore moved to dispense with the motion to adjourn, and vote for S. P. Thompson for membership. Adopted.

The rules being suspended, Dr. Thompson was declared a member.

Adjourned to meet at 2 o'clock P. M.

AFTERNOON SESSION.

The association met at 2 o'clock, pursuant to adjournment, Dr. Cunningham, the newly elected President, in the chair.

Prof. Moore presented the name of W. P. Crowell, who was unanimously elected a member.

"Filling teeth with heavy and light mallet," the first subject for discussion, was now declared in order.

Prof. Moore made some remarks in favor of the heavy mallet.

Prof. Chase gave his experience with mallets of different weights, and concluded a seven or eight ounce mallet preferable.

Dr. Knapp has used all kinds, and settled upon a heavy lead one.

Dr. Hunt next spoke in favor of the heavy mallet.

Dr. Snyder uses exclusively the elastic wooden mallet.

Drs. Scott, Palmer, Burt and others, all advocated heavy mallets.

On motion the subject was passed, and the second subject, "Materials for filling teeth," taken up.

Dr. Knapp uses Watts' foil, Nos. 3, 4, 10, 20, 30 and 60. Makes the main part of the filling with No. 4.

Dr. Hunt uses oxychloride of zinc, cement, amalgam and Johnston's Nos. 4 and 60, soft foil, principally No. 60.

Dr. Palmer uses gold, tin, Hill's stopping and oxychloride. *Nothing else.* Tin for children's teeth.

Prof. Chase advocates tin in place of amalgam in all approximal cavities. Thinks the amalgam of the present day more objectionable than the old fashioned way of preparing it. Uses gold for most cases, No. 4 to 6, adhesive, 4 to 8 soft foil (Abbey's). For building up, 20 to 60.

Prof. Moore advocates gold, and thinks cement plombe far superior to oxychloride. Has seen fillings after six months use firm and in good condition.

On motion the subject was here closed, and the third subject, "The expediency of building up cutting surfaces of incisors when worn away by mastication," was taken up.

Prof. Moore stated a case contemplated in the question, and as he had adopted the practice, wished to ask the views of other members.

Dr. Hunt gave a case of interesting and elaborate character, that resulted very satisfactorily to the patient and himself.

On motion adjourned to meet at half past 7 o'clock, P. M.

### EVENING SESSION.

The association was called to order at 7½ o'clock by the president.

Minutes of afternoon session read and approved.

Dr. Knapp spoke upon the subject in order, and gave an account of successful practice.

Prof. Chase spoke in approving terms of the practice.

Dr. Palmer also gave interesting accounts of cases in his practice, and continued at length with a full description of the *modus operandi*, kinds of foil, etc.

On motion of Prof. Moore the subject under discussion was dispensed with.

### CLINICS.

Prof. Moore moved that to-morrow morning be devoted to clinical operations, and Dr. Palmer appointed to operate. Carried.

On motion adjourned to meet at 8 o'clock on Wednesday morning at the dental apartments of Drs. Moore and Burt, for the purpose of holding the clinic.

### SECOND DAY—MORNING SESSION.

Association met according to adjournment at 8 o'clock, A. M., in the rooms of Drs. Moore and Burt. Dr. Palmer being unable to obtain a case suitable to demonstrate his method of filling. Prof. Chase occupied the entire morning in performing a very difficult and protracted operation in filling, much to the interest and profit of all who witnessed.

### AFTERNOON SESSION.

Association met at the regular place at 2 o'clock, P. M.. President Cunningham in the chair.

On motion the reading of the minutes was dispensed with.

On motion the regular order of business was dispensed with, and Dr. Palmer invited to occupy the afternoon in exhibiting and explaining his charts, casts and diagrams. With these many of the profession are acquainted. To those who have never seen them, a poor idea can be given by a description. The lessons learned from him are what none can afford to lose. He stands at the head as a reformer in dental practice. Taking *nature* for his model, all the wasted lines are by his skill remoulded into form. The association during the entire afternoon gave the closest attention to Dr. Palmer's illustrations and remarks, feeling that they had at this meeting received the greatest good that could possibly be bestowed upon them. After some formal business attending the close of the session, the association, on motion of Prof. A. M. Moore, adjourned to meet at Ft. Wayne, on the third Tuesday in March, in 1872.

At the solicitation of Prof. Moore, Dr. Palmer remained one day after the adjournment. When at the office of Drs. Moore and Burt, in the presence of a number who remained to witness, he demonstrated by operations on the natural teeth, his *perfect system* of filling teeth.

SENECA B. BROWN, *Secretary.*

